# Mixing Signals: Molecular Turn Ons and Turn Offs for Innate γδ T-Cells

**DOI:** 10.3389/fimmu.2014.00654

**Published:** 2014-12-18

**Authors:** Vasileios Bekiaris, John R. Šedý, Carl F. Ware

**Affiliations:** ^1^Infectious and Inflammatory Disease Center, Sanford-Burnham Medical Research Institute, La Jolla, CA, USA

**Keywords:** BTLA, dermatitis, γδ T-cell, IL-7, lymphotoxin, RORγt

## Abstract

Lymphocytes of the gamma delta (γδ) T-cell lineage are evolutionary conserved and although they express rearranged antigen-specific receptors, a large proportion respond as innate effectors. γδ T-cells are poised to combat infection by responding rapidly to cytokine stimuli similar to innate lymphoid cells. This potential to initiate strong inflammatory responses necessitates that inhibitory signals are balanced with activation signals. Here, we discuss some of the key mechanisms that regulate the development, activation, and inhibition of innate γδ T-cells in light of recent evidence that the inhibitory immunoglobulin-superfamily member B and T lymphocyte attenuator restricts their differentiation and effector function.

## Introduction

**Figure 1:**
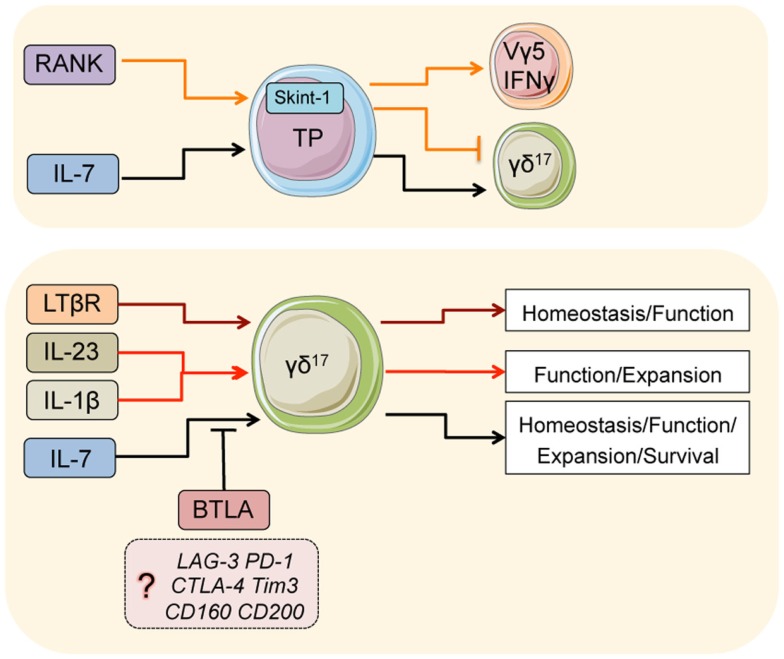
**Major pathways that regulate γδ^17^ T-cells**. Signals in thymic progenitors (TP): during development, RANK co-ordinates Skint-1 expression, which regulates the fate decision of thymic progenitors into γδ^17^ or Vγ5/IFNγ γδ T-cells. IL-7 is critical for the development of γδ^17^ cells from thymic progenitors. In the adults, IL-7 is also critical for the normal homeostasis, function, expansion, and survival of adult γδ^17^. BTLA (and perhaps other inhibitory receptors) suppress overt homeostatic proliferation and hyperactivation in part by regulating IL-7 responsiveness. LTβR is critical for normal homeostasis and function of γδ^17^ T-cells in the adult, likely through differentiation of the microenvironment. IL-23 and IL-1β are both critical cytokines that initiate inflammatory γδ^17^ responses.

The ability to generate antigen receptor diversity by somatic recombination evolved approximately 500 million years ago (1) and became the founding biological property of what we now know as adaptive immunity. This evolutionary milestone provided our immune system with an innate and an adaptive arm that synergized for the fight against infection and the recognition of oncogenesis. Lymphocytes of the gamma delta (γδ) T-cell lineage are evolutionary conserved among species (2) and although they express rearranged antigen-specific receptors, a large proportion display innate properties. In the mouse, where innate γδ T-cells have been mostly studied, approximately 25% of lymph node γδ T-cells respond rapidly to cytokine stimuli similar to innate lymphoid cells (ILCs) and appear to have reduced T-cell receptor (TCR) signaling capacity (3). Innate γδ T-cells are characterized by the spontaneous and high expression of interleukin (IL)-17 (γδ^17^) as well as IL-22 and express functional Toll-like receptors (TLR) (4, 5). Importantly, IL-17 and IL-23 receptor (IL-23R) expression, which is critical for IL-22 induction, are turned on during embryonic development in the thymus strongly pointing toward a *bona fide* innate nature (6–8). Although a new interferon gamma (IFNγ)-producing innate γδ T-cell subset with no IL-17 potential has recently been described (3), this review will discuss briefly some of the key cytokines, cytokine receptors, and transcription factors (TFs) that regulate the development, activation, and inhibition of mouse innate γδ^17^ cells (Figure 1).

## IL-23 and IL-1β: Key Proinflammatory and Anti-Bacterial Mediators

Innate γδ^17^ cells localize mainly at barrier and mucosal surfaces such as the skin, gut, and lung (9) and within the lymph nodes, they position themselves in close proximity to the subcapsular sinus and interfollicular regions both of which specialize in the capture of antigen (10). Therefore, infectious and inflammatory stimuli can readily activate γδ^17^ cells either directly through TLR ligation or through cytokines such as IL-23 and IL-1β that are produced by local innate sensors.

IL-23 induces the expression of IL-17 and IL-22 as well as the transcription factor retinoid-related orphan receptor gamma-t (RORγt) in T-helper 17 (T_H_17) cells while at the same time promoting survival and cell proliferation (11). γδ^17^ cells express functional IL-23R as early as embryonic day E18 in the thymus (7), in contrast to CD4^+^ T-cells that upregulate the IL-23R upon T_H_17 differentiation (12). Although IL-23 or IL-23R has not been reported to be important for γδ T-cell development, they enhance the production of IL-17 and IL-22 and can promote cellular proliferation (3, 13). *In vivo* infectious and inflammatory models have shown that IL-23 can be important for the activation of the γδ T-cell response.

During imiquimod (IMQ)-induced psoriasis, genetic ablation of IL-23 or IL-23R results in a significant reduction of IL-17 production by γδ^17^ cells, diminished accumulation of these cells in the skin, and a subsequent decrease in inflammatory symptoms (14–16). In this model, IL-23 is produced locally in the skin by resident macrophage and dendritic cell (DC) populations that receive a combination of TLR and neuronal signals (15, 17, 18). The onset of experimental autoimmune encephalomyelitis (EAE), which is often used to model human multiple sclerosis, also depends to a certain extent on IL-23-driven IL-17 production by γδ T-cells (5, 19). More specifically, it has been shown that IL-23-activated γδ^17^ cells are important for optimal T_H_17 polarization (5) and the suppression of regulatory T-cell responses (19). In a mouse model of brain ischemic injury, absence of IL-23 also abrogated γδ^17^-induced inflammation (20). In addition to regulating inflammatory reactions, γδ^17^ cells and IL-23 have been linked with protection from a number of bacterial infections. Thus, cutaneous infection with *Staphylococcus aureus* triggers a γδ T-cell orchestrated IL-17 response that depends on the combined effects of IL-23 and IL-1β (21). Furthermore, infection with *Listeria monocytogenes* elicits an IL-23-driven γδ^17^ response that is important for bacterial clearance (22, 23), and the IL-23 pathway appears also to operate during γδ^17^ activation by *Mycobacterium tuberculosis* (24). Together, these data highlight the role of IL-23 in activating γδ^17^ cell-induced inflammatory responses, both to pathogens and in driving autoimmune disease.

Similar to IL-23, IL-1β has also been linked with IL-17-related immunity both in CD4^+^ T as well as in innate γδ T-cells. γδ^17^ cells constitutively express the IL-1 receptor and respond to *in vitro* IL-1β stimulation by rapid proliferation and upregulation of IL-17 (3, 5, 13). Interestingly, IL-1β appears to be important for IL-23-mediated γδ T-cell expansion and IL-17 production although the molecular mechanism is not yet understood (5, 13). Effective IL-1β signaling was critical for γδ T-cell activation and disease progression in the EAE model (5). However, during IMQ-induced psoriasis, usage of *Ilr1^-/-^* mice has resulted in conflicting conclusions. Whereas an earlier report presented no impact of IL-1β on either dermatitis or γδ^17^ activation (25), a more recent study showed that *Ilr1^-/-^* mice were consistently protected with severely compromised γδ T-cell responses (13). A key difference in the two studies was the site of inflammation: ear (no IL-1β effect) (25) versus dorsal epidermis (strong IL-1β effect) (13), suggesting that IL-1β may have site-specific regulatory roles, such as differential effects on resident stromal and epithelial cells or due to differences in lymphatic drainage.

## IL-7: Keeping the Balance between Homeostasis and Inflammation

IL-7 is one of the best-studied T-cell homeostatic cytokines. IL-7 deficiency is associated with lymphopenia and dysfunction of naïve and memory T-cell subsets (26). IL-7 is essential for the development of γδ T-cells (27, 28) by regulating the survival of early thymic progenitors and by inducing V(D)J recombination within the TCR-γ locus (29, 30). Further experiments have shown that in addition to its developmental role, IL-7 supports the homeostatic proliferation of γδ T-cells (31). Although IL-7 is strongly associated with signaling via the signal transducer and activator of transcription 5 (STAT5) (32), it has been shown to induce STAT3 phosphorylation in diverse lymphocyte populations such as thymocytes (33), B-cell progenitors (34), and γδ T-cells (35). STAT3 is a critical component of the IL-23 and IL-6 signaling pathways, which are important for the differentiation of CD4^+^ T-cells into the T_H_17 lineage (11, 36), in part by antagonizing STAT5 (37). Of the γδ T subsets, IL-7 was found to preferentially expand and activate innate γδ^17^ cells in a STAT3-dependent manner (35), although it sustained survival of all γδ T-cells (38).

We have recently demonstrated that in γδ^17^ cells, STAT5-mediated IL-7 signaling induces surface expression of the checkpoint receptor B and T lymphocyte attenuator (BTLA), which is necessary for their normal homeostasis and activation during skin inflammation (38). Blockade of IL-7 signaling itself has been shown to acutely diminish γδ^17^-driven dermatitis (35) while during viral hepatitis IL-7 co-operates with IL-23 to rapidly activate intrahepatic γδ^17^ cells and initiate inflammation (39). Whether IL-7-induced STAT5 and STAT3 phosphorylation operate in parallel, sequentially, or as mutually exclusive processes within the γδ^17^ population is unknown. However, γδ T-cells deficient in STAT3 display normal homeostatic responses (40) suggesting that at steady state STAT5 may have a dominant role.

In addition to its direct effects on γδ T-cells, IL-7 indirectly influences innate γδ T-cell development by promoting the generation of lymphoid tissues in part by inducing the expression of tumor necrosis factor (TNF) superfamily members. IL-7 is produced homeostatically in the developing thymus and lymph node anlagen (41) and has been shown to induce the expression of surface lymphotoxin-αβ (LTαβ) on resident embryonic lymphoid tissue inducer (LTi) cells (42). LTαβ expressed by LTi interacts with the LTβ receptor (LTβR) in order to initiate lymph node development and organization (43, 44). Genetic ablation of LTβR results in the absence of all secondary lymphoid tissues in addition to disorganized splenic and thymic architecture (45, 46). Several members of the TNF superfamily have been shown to directly regulate γδ T-cell development, homeostasis, and function, as outlined below.

## Lymphotoxin and the TNF Network: Critical Regulators of Innate γδ T-Cells

Innate IL-17 producing γδ T-cells as well as Vγ5 (Vγ3 in Garman nomenclature) expressing cells that colonize the skin as resident dendritic epidermal T-cells (DETCs) are strictly dependent on the embryonic microenvironment (8, 47). Thus, adult progenitors cannot reconstitute either of the aforementioned populations even if they are provided with a fetal thymus suggesting the need for embryonic-only progenitors (8). Thus, the fetal thymus contains fully functional γδ^17^ cells that develop between E15–18 (8). The development of these cells is intimately associated with the TNF superfamily since as early as E15 Vγ5^+^ progenitors express the TNF ligand RANKL (receptor activator of NF-κB ligand) and condition the thymic medulla to upregulate Skint-1 (48), an immunoglobulin (Ig) superfamily protein that is necessary for the development of Vγ5 cells (49–51). Interestingly, in Skint-1 deficient animals, Vγ5 cells are reprogramed into a γδ^17^-like phenotype with severely reduced IFNγ production (52). This suggests that innate γδ^17^ T-cells are likely to represent the default differentiation pathway of most γδ T-cell progenitors pre-Skint-1 selection. This is in line with the evolutionary evidence that IL-17-producing γδ T-cells are conserved between non-jawed vertebrates and human beings (2) while Skint-1 and related genes (e.g., Btn1a1) are highly restricted to mammals (www.ensembl.org).

In addition to RANK, LTβR has also been linked with the development and functional maturation of γδ T-cells. Early reports showed that γδ T-cells can acquire LTβR expression in the thymus, and that activation of these receptors by LTαβ- and LIGHT-expressing double-positive (DP) thymocytes drives maturation of γδ T-cells assessed by the production of IFNγ (53). However, the expression of IL-17 or other γδ^17^-related properties was not evaluated. The authors suggested that LTβR-induced maturation likely occurred at the late stages of thymic development when DP cells predominate. Given that γδ^17^ T-cells develop during early embryonic life (8), one scenario to explain these findings is that during thymic development the LTβR pathway in part regulates the IFNγ potential of γδ T-cells, presumably following Skint-1 selection. In agreement with this argument, the TNF receptor CD27 is required by thymic progenitors to induce the innate IFNγ-related differentiation program and to sustain expression of LTβR (7). Thus, while CD27 deficient animals retain an intact γδ^17^ compartment, they showed a marked reduction in IFNγ and LTβR expression (54). These results predict that LTβR signaling is not absolutely necessary for γδ^17^ development and function, although mice deficient in LTβR or its ligands had very few IL-17-producing γδ T-cells in the spleen and thymus (55). Mice lacking the NF-κB TFs RelA and RelB also showed reduced IL-17-producing γδ T-cells (55). Since the NF-κB pathway is central to TCR signaling and T-cell development (56), low IL-17 production might be reflective of impaired TCR stimulation rather than loss of LTβR signals. Furthermore, lack of lymph nodes in LTβR deficient mice (45) may relocate γδ^17^ cells to the skin or intestine and thus explain their reduced numbers in the spleens. Importantly, loss of LTβR results in abnormal thymic organization and maturation of the medullary epithelium (46, 57), which may negatively affect γδ^17^ T-cell development. Alternatively, organized secondary lymphoid tissues may be important for the survival and steady-state turnover of γδ^17^ cells. Of note, LTβR has been shown to participate in the production of IL-7 by fibroreticular stromal cells in the lymph node (58), which might explain why deficiency in LTβR can result in reduced γδ^17^ responses.

In addition to its involvement in stromal cell development, LTβR is expressed on tissue resident DCs and macrophages (59) both of which have been linked with the IL-23-mediated activation of γδ^17^ T-cells, whether this is in the context of skin (15, 17) or brain inflammation (5). Notably, LTβR regulates the homeostasis of DCs (60, 61) and can directly induce their production of IL-23 (62). Interestingly, an LTβR-LTαβ interaction has been linked with the production of IL-22 by intestinal ILCs (63, 64) raising the possibility that a similar mechanism may be in place at sites where γδ^17^ cells preferentially localize, such as the skin.

## BTLA and Inhibitory Receptors: Putting the Brakes On

In human beings, herpesvirus entry mediator (HVEM) interacts with the two TNF ligands LIGHT (shared with LTβR) and soluble LTα, and the Ig superfamily members CD160 and BTLA. BTLA is an inhibitory receptor with an immunoreceptor tyrosine inhibitory motif (ITIM) that has been shown to interact with the Src homology 2 (SH2)-domain containing protein tyrosine phosphatase 1 (SHP1) and SHP2 and to inhibit T-cell activation (65–67) upon interacting with HVEM, its only identified ligand thus far (66, 68, 69). In addition to its inhibitory role in T-cell responses, BTLA was shown to prevent overt TLR stimulation in DCs (70) and to diminish cytokine production by natural killer T (NKT) (71) and follicular T-cells (72) suggesting a regulatory role both in adaptive and innate immunity.

**Figure 2:**
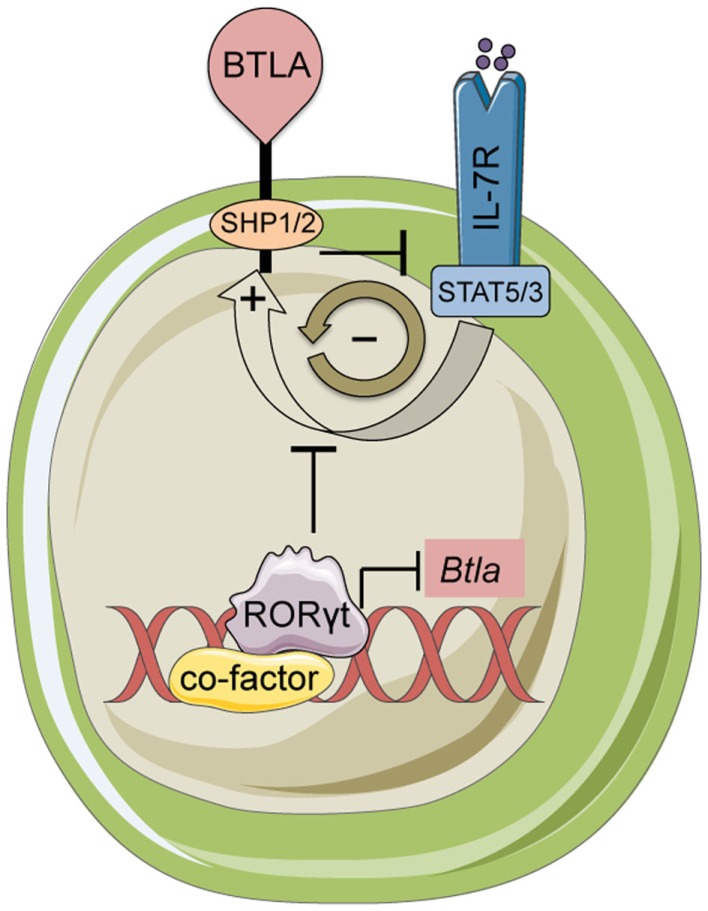
**BTLA and RORγt mediated control of γδ^17^ T-cells**. BTLA expression limits inflammatory responses and homeostasis of γδ^17^ cells by antagonizing IL-7 signaling. In turn, IL-7 induces BTLA expression creating a negative feedback loop. The transcription factor RORγt represses the *Btla* promoter limiting the expression level of BTLA. This regulatory loop maintains BTLA expression at very low levels on the cell surface in resting γδ T-cells.

BTLA and HVEM signal bi-directionally providing inhibitory signals in T-cells and survival signals in cells expressing HVEM (68). BTLA expression varies ~10^3^ fold among hematopoietic lineages, and co-expressed with HVEM forming a complex *in cis* that may contribute to homeostatic signaling (73). Constitutive surface expression of BTLA (74) implicates a unique ability among inhibitory receptors to sustain the homeostatic balance of T-cells (75) and DCs (61). Similarly, our recent data showed that BTLA is necessary to inhibit homeostatic expansion and activation of lymph node and skin resident γδ^17^ T-cells (38). γδ^17^ but not other γδ T-cell subsets deficient in *Btla* were hyperresponsive to IL-7 stimulation suggesting that BTLA diminishes IL-7 receptor (IL-7R) signaling. Interestingly, IL-7 increased surface BTLA on γδ^17^ cells in a STAT5-dependent way revealing the presence of a negative feedback loop between IL-7 and BTLA (38) (Figure 2). Given the broad range of SHP1 and SHP2 targets (76), it is likely that these phosphatases can inactivate both STAT3 and STAT5 in response to IL-7. However, the exact molecular details of BTLA-mediated suppression of IL-7R or other γδ^17^-expressed cytokine receptors are currently not known.

Although there are numerous functional inhibitory receptors that have been reported on the surface of lymphocyte subsets either at steady state or after activation, there is little information regarding their role on innate or non-innate γδ T-cells. Several reports have mapped the expression of inhibitory molecules like programed death-1 (PD-1) (77, 78), lymphocyte activation gene-3 (LAG-3) (79), CD200 (80), Tim-3 (81), CD160 (82), and cytotoxic T lymphocyte antigen-4 (CTLA-4) (83) on human or murine γδ T-cells but the capacity to target these receptors using agonistic or antagonistic manipulation has in general not been addressed. Notably, we found that activating BTLA receptors using an agonistic antibody limited pathology in mice (38). Additionally, blockade of BTLA signaling enhanced activation of lymphoma-specific human Vγ9Vδ2 T-cells (84). Thorough investigation of the expression patterns and function of the different inhibitory receptors on innate γδ T-cells may provide promising targets for intervening when these lymphocytes need to be turned on or off. Currently, and in combination with its suppressive activity, BTLA appears to be a key targetable pathway for regulating innate γδ T-cells.

## Transcriptional Control: Is Everything Pre-Programed?

It is now well-appreciated that there is an extensive network of TFs that are expressed early in pre-committed progenitors and are necessary for the development, functional differentiation, and survival of all innate cells including γδ^17^ T-cells. A subset of these TFs control lineage specification, either through activating or repressing gene transcription. A number of TF mouse knockout lines result in the complete abolishment or severe reduction in the numbers of the γδ^17^ subset in the periphery and in the thymus. Thus, mice deficient for the high-mobility group (HMG) box TFs Sox13 and Sox4 show severe reduction of IL-17-producing γδ T-cells due to a differentiation block early on during development (85, 86), which correlates with high expression levels of Sox13 and Sox4 in γδ^17^-comitted T-cell progenitors (86–88). Interestingly, the function of Sox13 can be counteracted embryonically by Egr3, which drives the DETC differentiation program and IFNγ expression (52), while TCF1, another HMG box TF, suppresses γδ^17^ differentiation (86). Notch signaling turns on TCF1 (89), which can also induce expression of Hes1, another TF critical for the generation of γδ^17^ cells during embryonic differentiation (40). Interestingly, a subset of innate γδ T-cells has been shown to depend on the expression of promyelocytic zinc finger (PLZF), which is also required for the development of ILCs (90, 91). It remains to be seen whether PLZF is specifically required for the development of γδ^17^ cells.

Although, RORγt is necessary for the differentiation of T_H_17 cells (36), it is not essential for the development of γδ^17^ progenitors in the fetal thymus (40). However, consistent with its ability to bind to and transactivate the *Il17* promoter (92), RORγt is important for optimal IL-17 production (40). Interestingly, despite being developed, RORγt deficient γδ T-cells cannot persist in the periphery (40), suggesting a potentially critical role for RORγt in the homeostasis of adult γδ^17^ T-cells. This could be either cell-extrinsic or cell-intrinsic. RORγt is necessary for the development of all secondary lymphoid tissues (93). Thus, upon export in the periphery, γδ^17^ T-cells may not have the appropriate microenvironment in order to sustain homeostasis (cell-extrinsic). In the cell-intrinsic scenario, RORγt may be important for the survival of γδ^17^ cells by regulating the levels of the anti-apoptotic protein Bcl-xL (93). Our data have demonstrated that via its interaction with LxxLL containing nuclear co-factors RORγt can function as a transcriptional repressor and suppress expression of BTLA (38) (Figure 2). Therefore, an alternative cell-intrinsic hypothesis is that loss of RORγt results in aberrant expression of BTLA and perhaps other co-inhibitory receptors (such as LAG-3; Bekiaris/Ware, unpublished observations) leading to a sustained inhibition of homeostatic expansion.

## Conclusion

γδ^17^ and other γδ T-cell subsets comprise a unique family of lymphocytes that provides an innate powerhouse to the immune system. The innate nature of γδ^17^ cells is demonstrable by a number of key biological properties including rapid response to cytokines, functional maturation during embryogenesis, largely TCR-independent responses, and TF-dependent lineage commitment. Resolving the complex and fascinating biology of these cells has been breaking the Frontiers of Immunology for a number of years and has taught us a great deal about how lymphocytes develop and function. The continued knowledge of how all innate γδ T-cells work will certainly push forward these frontiers and perhaps allow us to develop tools in order to manipulate them for the treatment of human disease.

## Conflict of Interest Statement

The authors declare that the research was conducted in the absence of any commercial or financial relationships that could be construed as a potential conflict of interest.
